# Incidence of moral hazards among health care providers in the implementation of social health insurance toward universal health coverage: evidence from rural province hospitals in Indonesia

**DOI:** 10.3389/fpubh.2023.1147709

**Published:** 2023-08-17

**Authors:** Syafrawati Syafrawati, Rizanda Machmud, Syed Mohamed Aljunid, Rima Semiarty

**Affiliations:** ^1^Faculty of Medicine, Andalas University, Padang, Indonesia; ^2^Department of Community Medicine, School of Medicine, International Medical University, Kuala Lumpur, Malaysia; ^3^International Center for Casemix and Clinical Coding, Faculty of Medicine, National University of Malaysia, Cheras, Malaysia

**Keywords:** moral hazards, up-coding, readmissions, unnecessary admissions, fraud, physicians, coders, patients

## Abstract

**Objective:**

To identify the incidence of moral hazards among health care providers and its determinant factors in the implementation of national health insurance in Indonesia.

**Methods:**

Data were derived from 360 inpatient medical records from six types C public and private hospitals in an Indonesian rural province. These data were accumulated from inpatient medical records from four major disciplines: medicine, surgery, obstetrics and gynecology, and pediatrics. The dependent variable was provider moral hazards, which included indicators of up-coding, readmission, and unnecessary admission. The independent variables are Physicians' characteristics (age, gender, and specialization), coders' characteristics (age, gender, education level, number of training, and length of service), and patients' characteristics (age, birth weight, length of stay, the discharge status, and the severity of patient's illness). We use logistic regression to investigate the determinants of moral hazard.

**Results:**

We found that the incidences of possible unnecessary admissions, up-coding, and readmissions were 17.8%, 11.9%, and 2.8%, respectively. Senior physicians, medical specialists, coders with shorter lengths of service, and patients with longer lengths of stay had a significant relationship with the incidence of moral hazard.

**Conclusion:**

Unnecessary admission is the most common form of a provider's moral hazard. The characteristics of physicians and coders significantly contribute to the incidence of moral hazard. Hospitals should implement reward and punishment systems for doctors and coders in order to control moral hazards among the providers.

## 1. Introduction

A moral hazard refers to the possibility of consumers or health care providers abusing a system in order to maximize profits at the expense of other consumers, providers, or the financing community as a whole (1). A moral hazard occurs, for example, when an insured person spends an extra day in the hospital or pays for a procedure that would not have been purchased otherwise (2). In insurance industry, the phenomenon of moral hazard umbrella may be considered as fraud. Insurance fraud would not be possible without asymmetric information—and cheating on insurance companies is deemed immoral—it is referred to as a moral hazard (3). Health insurance fraud can be committed by medical providers, policyholders, or health insurers. Although anyone in the system is capable of committing fraud, healthcare providers are more likely than patients to do so (4, 5).

Some of the healthcare fraud schemes that are frequently discussed in the literature and used to develop fraud detection algorithms or analytics within regulatory entities are as follows: Diagnostic-Related Groups (DRG) creep, unbundling and fragmentation of procedures, up-coding of services, phantom billing, providing excessive services that are not required, kickback schemes, billing for mutually exclusive procedures, duplicate claims and intentional billing errors (6). A number of studies in the world has proven that, provider moral hazard among providers did exist in hospital services (7).

Moral hazard has preoccupied health economics and U.S. health policy for half a century (8). When Medicare providers' payment patterns changed to a prospective Diagnostic Related Groups (DRG) system in the United States, hospitals raised the patient's disease code to a higher level (up-coding). It is aimed at getting the hospital's finance higher than they should be. In private hospitals, the response was stronger. Up-coding or code creeps also occurs in independent medical practices where there is an increase in claim payments, 2.2% from what it should be in 1 year. Hospitals respond to changes in payment patterns by changing the intensity of service provided to patients, severity levels, and market share (7, 9, 10).

Alonazi (11) conducted an audit of the Saudi healthcare system and found the official documents contain the details of various moral hazard measures. Berta et al. (12) examined several types of deviant in Italian hospitals and linked them to hospital efficiency. Deviations in question include up-coding, cream skimming, and readmissions. Debpuur et al. (13) found that the form of moral hazard in the Northern Ghana National Health Insurance is diagnosing simple malaria with complicated malaria, exaggerating the provision of drugs and health services to patients, asking for payments for services that are not provided, and increasing the number of patients receiving health services.

World Health Organization (WHO) estimates the annual global health care expenditure is US$ 5.7 trillion (2008). Each year, 7.29% of that, or an estimated US$ 415 billion, is lost to fraud and errors. South Africa's healthcare system is defrauded between 4 million and 8 billion US Dollars annually. In the UK in 2008–2009, about 3% of National Health Services (NHS) fees were lost to fraud (14). The Centers for Medicare and Medicaid Services (CMS) spent $1.1 trillion on health coverage for 145 million Americans in 2016, $95 billion of which was improper payments related to abuse or fraud (15).

According to a 2009 study, 19.6% of 11.8 million Medicare beneficiaries who were hospitalized from 2003 to 2004 were readmitted within the first month of their hospitalization, costing an estimated $41 billion per year (16). According to Geruso and Layton (2020), upcoding could have cost Medicare $10.5 billion in 2014, or $640 per Medical Advantage enrollee (17). Between July 2009 and June 2010, 139 patients were admitted and treated for preterm labor at a level III center, but none of them delivered preterm. Total hospital charges for the management of these patients were $1,018 589. Unnecessary admissions and treatments for threatened preterm labor are part of clinical practice and contribute to exploding healthcare costs (18).

Indonesia is the world's largest country that aims to achieve Universal Health Coverage through the National Health Insurance. However, the health financing fund was claimed to contribute to budget deficit from USD 200,000,000 in 2014 to USD 450,000,000 in 2016. The moral hazard of health providers has been blamed as one cause of the deficit. In 2015, there were around 175 thousand claims from health services managed by National Health Insurance Administration Agency or recognized by name *BPJS Kesehatan* with a value of 27 million dollars that was detected as fraud, and up to now there have been 1 million claims detected. Nevertheless, so far no independent study was done to assess the real incidence and cause of moral hazards in the National Health Insurance of Indonesia (7, 19, 20). This research can contribute to improve the implementation of Indonesian National Health Insurance by providing scientific evidence on the existence and sources of moral hazards among providers. This will allow the relevant parties to forecast the events and take preventive actions in the future.

On the other hand, systematic review conducted by Pongpirul and Robinson (21) stated that the actors of moral hazard in hospitals could be classified into three categories; those are hospital management, clinicians, and coders (21). However, no study has shown the relationship of moral hazard with the type of hospital, physician, coder, and patient characteristics. The hypothesis we seek to test is that there is a relationship between the characteristics of physicians (age, gender, and specialization), coders (age, gender, education level, number of certificates, and length of service), and patients (age, birth weight, LOS, discharge status, and the severity of the illness) with the incidence of moral hazards. This study aims were to identify the incidence of moral hazards and determinant factors such as physicians, coders and patients characteristics in the implementation of national health insurance in Indonesia.

## 2. Methods

### 2.1. Design

We conducted a cross-sectional study on representative Class C hospitals to undertake medical record analysis in West Sumatera Province, Indonesia.

#### 2.1.1. Population

In this study, the population consisted of medical records from inpatients in class C hospitals in West Sumatra. According to data from the Ministry of Health, class C hospitals are the most common type of hospital in West Sumatra. According to data from the Health District Office West Sumatera Province, there are 38 Class C hospitals, 15 of which are government-owned and 23 of which are private (22). A Class C hospital is one that offers four basic medical specializations: surgery, obstetrics and gynecology (OBGYN), pediatrics, and internal medicine.

#### 2.1.2. Sample

A cluster random sampling technique was used to choose the hospitals. Cluster random sampling divides the population into clusters/classes, with the assumption that each class/cluster already has the trait/variation under study. In this study, 6 (six) Class C hospitals were selected, consisting of three government hospitals and three private hospitals. In accordance with the sample size calculation, the minimum sample size for this study is 360 medical records.

**Figure 1:**
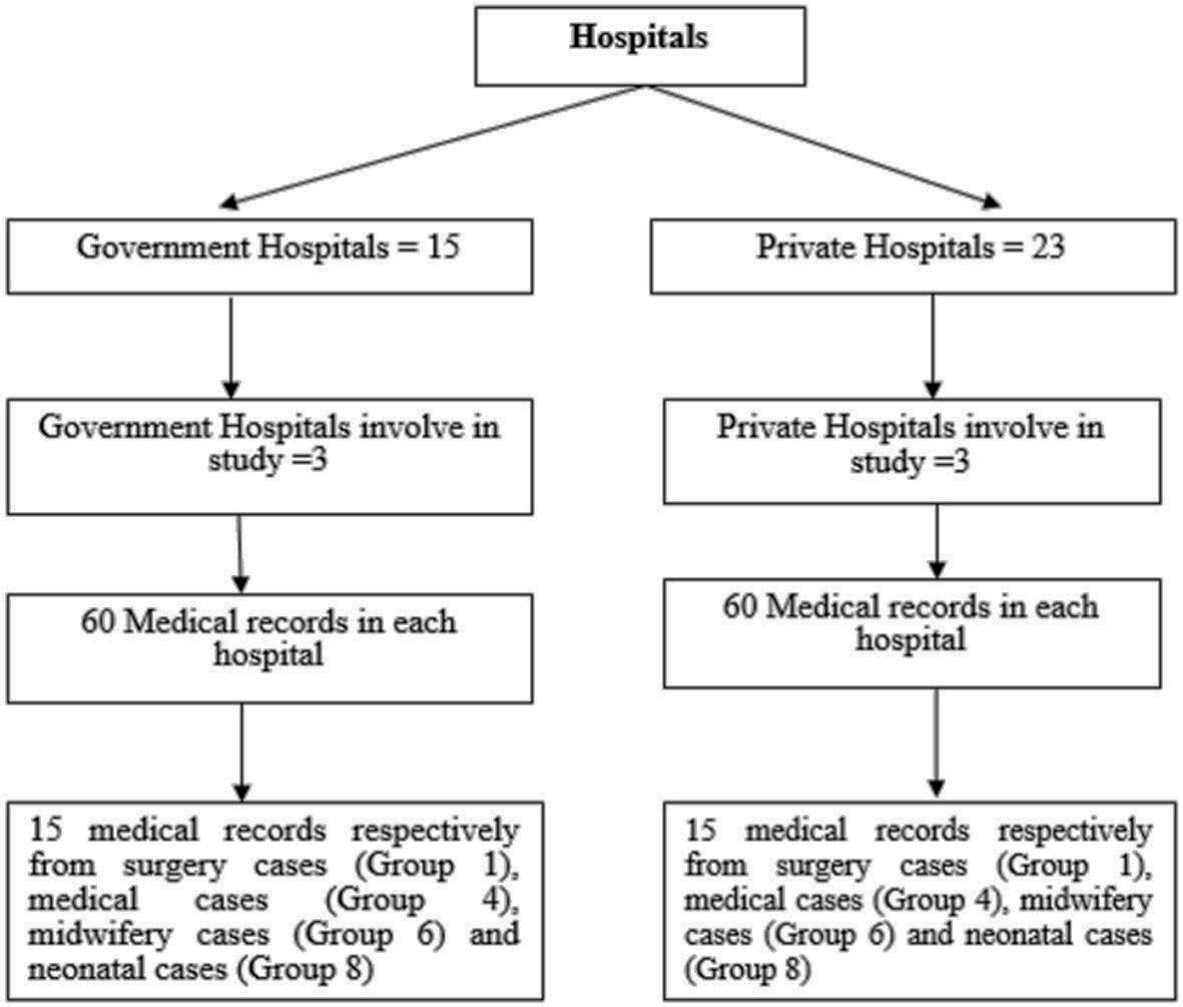
Sample selection process.

Six hospitals receive an equal quantity of samples. In each hospital 60 medical records were selected. The 60 patient medical records will be split into four primary groups of INA-CBG internal disease cases: surgical cases (Group 1), medical cases (Group 4), delivery cases (Group 6), and neonatal cases (Group 8). The number of medical records obtained for each case was 60/4, i.e., 15 medical records per case. The sampling method in this study is shown in Figure 1.

The sample inclusion criteria include medical records which have complete data on 14 casemix variables, including: Patient Data (1). Identification: Patient name, Medical Record Number; (2). Age in years; (3). Age in days; (4). Gender; (5). Date of Birth; (6). Birth Weight (for neonates), Admission Data (7). Date of hospital admission (8). Discharge Date (9). Length of stay (LOS) (10). Discharge Disposition Clinical Data (11). Primary Diagnosis; (12). Secondary Diagnosis; (13). Primary Procedure; (14). Secondary Procedure. Sample exclusions criteria are medical records that are not found, damaged, or cannot be read by an independent coder. Data collection was carried out in January–June 2018.

### 2.2. Data collection

The data were collected by independent reviewers, namely several senior medical record professionals who did not work in the selected hospitals and have a minimum of 5 to 10 years of experience as coders. Furthermore, the qualification for selecting independent reviewers is that they have attended INA-CBG coding training on a national scale five times or more. The reviewer's job is to go over the medical records of the patient who were chosen as samples. The function of an independent reviewer is to code the patient's illness based on the information in the medical records. Because the independent coder is not involved in the service and management processes at the hospital under review, we consider the results of this coding to be the gold standard for medical coding. Furthermore, the reviewers gathered secondary data in the form of the characteristics of the coder, clinician, and patient, which were the study's independent factors.

### 2.3. Study outcomes

#### 2.3.1. Moral hazard

Three indicators of moral hazards are used in this study: up-coding, readmission and unnecessary admission. These variables were derived from systematic review and a pilot study to identify the main moral hazard indicators in hospitals.

Up-coding is the mismatch between the diagnosis code and procedure written in the medical records which causes an increase in hospital reimbursement (7, 23–25). In this study, an Independent Senior Coder (ISC) reviewed each of the medical records. The codes were entered into INA-CBGs software to determine the hospital tariff. Furthermore, researchers gathered data on the results of medical coding executed by the hospital coder (original codes) and their tariff. The tariff based on the original codes was compared with the codes from ISC. If the tariff from the hospital coder's work is higher than ISC codes, then the case is considered as up-coding.

Readmission is an event of patient service where the same discharged inpatient is brought back for hospitalization to undergo the same disease treatment after a period of < 30 days (26). In this study, we reviewed the medical records and if the is hospitalized in the same hospital for the same disease they had previously been treated and discharged < 30 days, then it is classified as a readmission case.

Unnecessary admission is a hospitalization case where there is no significant reason for the patient to be treated when they were first admitted to the hospital (26). In this study, unnecessary admission is defined as any admission with a length of stay (LOS) of 2 days and below, and the patient is discharged well (not dead). However, admissions that ended in death are not considered unnecessary admissions.

#### 2.3.2. Characteristics of patients

Patient data is an important factor to determine INA-CBGs tariff. Patient data consist of demographic data, admission data, and clinical data. So far, there were no studies that look at the relationship between patient variables with moral hazard. Patient variables in this study were patients' age, birth weight, LOS, discharge status, and the severity of the patient's illness.

#### 2.3.3. Characteristics of coders

A coder is a person who assigned the diagnoses and procedure codes and enters the minimum data set into INA-CBGs software in order to produce INA-CBGs tariff. The coder's qualification is very decisive for coding quality. Coders' characteristics in this study were age, gender, education level, number of certificates, and length of service.

#### 2.3.4. Characteristics of physicians

In terms of health care provided in hospitals, a physician is a person who has responsibility for patient care. The physician also has the potential to perform moral hazard by increasing admission volume, changing the intensity of care, and exaggerating (21). Physician variables in this study were age, gender, and specialization (medical, surgical, OBGYN, pediatric).

### 2.4. Statistical analysis

The incidence of moral hazard and characteristics of doctors, coders, and patients are described in the frequency distribution table. Incident moral hazard consists of up-coding, readmission, and unnecessary admission. The physician's characteristics include the physician's age, physician's gender, and physician's specialization (medical, surgical, OBGYN, and pediatric). The characteristics of the coder consist of the coder's age, coder's gender, education level of coders, the number of coder's certificates, and coder's length of service. Patient characteristics consist of patients' age, birth weight, LOS, discharge status, and the severity of the patient's illness.

We used multilevel logistic regression analysis to examine the contributions of characteristics of the patient, coder, and physician to the incidence of moral hazards. The following multilevel model was used (27).


$$\begin{array}{l} \frac{P\left(y=1|x_{ij},\eta_{0j}\right)}{P\left(y=0|x_{ij},\eta_{0j}\right)}=\beta_{0}+\beta_{1}+\ldots \beta_{\text{k}}X_{\text{kij}}+\eta_{0\text{j}}+e_{\text{ij}} \end{array}$$

In the presence of more than one explanatory variable, logistic regression is used to calculate the odds ratio. With the exception that the response variable is binomial, the approach is quite similar to multiple linear regression. The impact of each variable on the odds ratio of the observed event of interest is the result (28).

### 2.5. Ethics approval

Ethical approval for this study was obtained from Faculty of Medicine Andalas University (No. 052/KEP/FK/2018).

## 3. Results

### 3.1. Incidence of moral hazard

Detailed indicators of moral hazard are presented in Table 1. The most common type of moral hazard was possible unnecessary admission (17.8%), followed by up-coding (11.9%) and readmission (2.8%).

**Table 1:** Incidence of moral hazard.

**Indicators of moral hazard**		**Numbers**	**%**
Up-coding	Yes	43	11.9
	No	317	88.1
Readmission	Yes	10	2.8
	No	250	97.2
Unnecessary admission	Yes	64	17.8
	No	296	82.8
Total		360	100

Unnecessary admissions were up to 4.2% more common among neonates group. Meanwhile, deliveries group dominated up coding cases by as much as 2.8%. Readmissions were more common in neonatal groups and female reproductive system groups. The full results can be seen in Table 2.

**Table 2:** Percentage of moral hazard types based on casemix main group.

**Moral hazard**	**Casemix main groups**	**Total**	**%**	**Explanation**
Up-coding (43)	O	10	2.8	Deliveries group
	W	6	1.7	Female reproductive system groups
	P	4	1.1	Newborns and neonates groups
	K	4	1.1	Digestive system group
	G	3	0.8	Central nervous system groups
	I	3	0.8	Cardiovascular system groups
	L	3	0.8	Skin, subcutaneous tissue and breast group
	M	3	0.8	Musculoskeletal system and connective tissue groups
	D	2	0.5	Haemopoeitic and immune system groups
	C	1	0.3	Myeloproliferative system and neoplasms groups
	E	1	0.3	Endocrine system, nutrition and metabolism groups
	J	1	0.3	Respiratory system groups
	N	1	0.3	Nephro-urinary system groups
	U	1	0.3	Ear, nose, mouth and throat groups
*Readmission* (10)	P	2	0.5	Newborns and neonates group
	W	2	0.5	Female reproductive system groups
	G	1	0.3	Central nervous system groups
	I	1	0.3	Cardiovascular system group
	J	1	0.3	Respiratory system groups
	K	1	0.3	Digestive system group
	L	1	0.3	Skin, subcutaneous tissue and breast groups
	U	1	0.3	Ear, nose, mouth and throat groups
Possible unnecessary admission (64)	P	15	4.2	Newborns and neonates group
	O	14	3.9	Deliveries group
	U	10	2.8	Ear, nose, mouth and throat groups
	M	6	1.7	Musculoskeletal system and connective tissue groups
	G	4	1.1	Central nervous system groups
	H	3	0.8	Eye and adnexa groups
	L	3	0.8	Skin, subcutaneous tissue and breast groups
	W	3	0.8	Female reproductive system groups
	D	2	0.5	Haemopoeitic and immune system groups
	K	2	0.5	Digestive system group
	I	1	0.3	Cardiovascular system groups
	N	1	0.3	Nephro-urinary system groups

### 3.2. The characteristic of physicians, coders, and patients

Table 3 illustrates the characteristics of physicians, coders and patients. The average age of physicians was 41 years old. It means that the physicians involved in this study were mostly young. Similarly the average age of coders was 31.32 years old. Meanwhile, the average age of patients was 26, 49 years old, and average LOS was 4 days.

**Table 3:** Characteristics of physicians, coders, and patients.

**Variables**	**Mean (±SD)**	**Median**	**Max**	**Min**
Physicians; age	41.04 (±9,037)	39	72	31
Coders; age	31.32 (±5,244)	30	40	26
Patients' age	26.49 (±22,387)	25.5	86	0
LOS	4.18 (±2,213)	4	20	1

Most of physicians' were male and one third of them were specialized in OBGYN (31.4%). Half (50.8%) of coders have had < 4 years work experience and more than half coder (67.5%) have at less only one INA CBG coding training. Most of patients (71.4%) are females, with the discharge status dominated discharge home (93.6%). Most of the infant patients have not experienced low birth weight (84.6%) as illustrated in Table 4.

**Table 4:** Characteristics of physicians, coders, and patients (categorical data).

**Variabel**	**F**	**%**
**Physician**		
Physician's sex		
Male	244	**67.8**
Female	116	32.2
Physician's specialization		
Surgery	78	21.7
Medical (Internal Medicine, Ophthalmology, Cardiology, ENT (Ear, Nose and Throat), Pulmonology	67	18.6
Obstetric and genecology	113	**31.4**
Pediatric	102	28.3
**Coder**		
Coder's Sex		
Male	0	0
Female	360	**100**
Coder education		
Lower than diploma	0	0
Diploma and higher	360	**100**
Number of coder's training		
One and none	243	**67.5**
More than one	117	32.5
Coder length of services		
Less than 4 years	183	**50.8**
More than 4 years	177	49.2
**Patients**		
Patient's sex		
Male	103	28.6
Female	257	**71.4**
Birth weight (neonates)		
Low birth weight	14	15.4
Normal	77	**84.6**
Discharge status		
Discharge home	337	**93.6**
Transferred to other hospitals	11	3.1
Discharge against medical advice	6	1.7
Death	6	1.7
Primary diagnosis		
A00-B99	8	2.2
C00-D48	25	6.9
D50-D89	6	1.7
E00-E90	6	1.7
H00-H59	2	0.6
I00-I99	23	6.4
J00-J99	22	6.1
K00-K93	32	8.9
L00-L99	5	1.4
M00-M99	6	1.7
N00-N99	10	2.8
O00-O99	105	**29.2**
P00-P96	89	**24.7**
Q00-Q99	2	0.6
R00-R99	6	1.7
S00-T98	8	2.2
Z00-Z99	5	1.4
Primary procedure		
No procedure	139	**38.6**
01–05	1	0.3
06–07	1	0.3
08–16	3	0.8
21–29	17	4.7
30–34	2	0.6
35–39	2	0.6
40–41	1	0.3
42–54	21	5.8
55–59	1	0.3
65–71	8	2.2
72–75	89	**24.7**
76–84	19	5.3
85–86	21	5.8
87–99	35	9.7
Severity level of illness		
Severity level 3	26	7.2
Severity level 2	57	15.8
Severity level 1	277	**76.9**
**Total**	**360**	**100**

The bold values indicate the highest percentage of each variable.

It could be seen from multivariate analysis that physician's age, physicians' specialization, coders' length of service, and LOS have significant relationship with incidence of moral hazard. The most remarkable influence on moral hazard cases is physicians specialization variable. To put it simply older physicians, medical specialization, coders with less length of service, and long LOS had a significant relationship with the incidence of moral hazard. The full results can be seen in Table 5.

**Table 5:** Multiple logistic regression analysis of factors influencing moral hazards.

**Variabel**	**B**	**S.E**.	**Wald**	***p*-value**	**POR**	**95.0% C.I. for EXP(B)**
Physician's age	0.036	0.018	4.054	0.044	1.037	1,001–1,073
**Physician's specialization**						
Surgery	0.752	0.356	4.459	0.035	2.121	1.056–4.262
Medical	0.864	0.377	5.257	0.022	2.373	1.134–4.262
Obstetric and gynecology	0.219	0.338	0.421	0.517	1.245	0,642–2,415
Pediatric					1	
Coder length of services	0.805	0.255	9.942	0.002	2.237	1,356–3,691
LOS	0.222	0.069	10.397	0.001	1.249	1,091–1,430

## 4. Discussion

Indonesia offers significant funding for JKN implementation. According to the *BPJS Kesehatan* financial report, health insurance expenses totalled 6.364 billion US dollar in 2018. Several rules to prevent moral hazard or fraud have also been implemented, such as the release of Regulation of the Minister of Health no. 36 of 2015 on hospital fraud prevention. However, no research has been conducted to demonstrate the efficiency of these preventative measures against moral hazard situations in hospitals.

This study found the incidence of unnecessary admission was the highest moral hazard indicator at 17.8%. This finding is higher than in other studies elsewhere. According to Mosadeghrad and Isfahani (30) research on the measurement of unnecessary patient admissions in Iranian hospitals, 2.7% of hospital admissions were considered unacceptable and unnecessary. The highest unnecessary patients' admissions in hospital were 11.8%, and the lowest were 0.3%.

Unnecessary admission is “an admission that provides no significant benefit to the patient or provides a benefit that could have been obtained at a lower level of care (31). In this study, the term “unnecessary admission” refers to patients who are hospitalized for 1 to 2 days with a non-dead discharge status. Unnecessary admissions mostly occurred in the Neonatal Group and Deliveries Group. Other studies elsewhere on unnecessary admissions were mostly found in the emergency departments (32–34).

A variety of patient-related factors (e.g., age, disease severity, method of payment, and route and time of admission), physicians, and the hospital and its diagnostic facilities and technology influence the unnecessary admission of patients to the hospital. Unnecessary hospitalization increases nosocomial infections, morbidity, and mortality, and reduces patient satisfaction and hospital productivity (35–39).

Previous researchers proposed several strategies for reducing avoidable hospital admissions, including expanding the primary health care network, reducing hospital beds, implementing an effective and efficient patient referral system, using a fixed provider payment method, promoting residential and social services care at the macro level, establishing a utilization management committee, using the appropriateness evaluation protocol, establishing short-stay units, and establishing a patient referral system (30, 33, 40, 41).

Indonesia has implemented several of these strategies in its health care system, such as implementing a patient referral system. The National Health Insurance Administration Agency has a tiered referral system that must be implemented by health insurance participants, social health insurance companies, and health facility providers. This tiered referral system operates on a hierarchical basis, beginning with primary health facilities (the closest to the community) and progressing to secondary and tertiary health facilities. Referral to second-level health facilities can only be administered by a first-level health facility (42).

With the existence of a referral system, where there are criteria such as health services in primary health care facilities that can be referred directly to tertiary health care facilities only for cases that have been diagnosed and a treatment plan has been established, a repeat service and is only available in tertiary health care facilities, reducing the incidence of unnecessary admissions because there is a system that must be followed, unnecessary admissions will be reduced. This system is strengthened by the existence of a policy that states that if a health facility does not adopt a referral system, *BPJS Kesehatan* will conduct re-credentialing on the health facility's performance, which may have an impact on future collaboration.

However, hospitals must strengthen management to avoid unnecessary admissions by establishing a utilization management committee and implementing the appropriate evaluation protocol.

The second type of moral hazard found in this study was up-coding (11.9%). In Germany, up-coding occurs at 1% of inpatients' payments (29). Another study found a fairly high incidence of up-coding, estimating that 18.5% annual reimbursed claims for Present on Admission (POA) infections were up-coded hospital-acquired infections (HAIs) (43).

Hospitals in Germany have up-coded at least 12,000 premature babies and received additional reimbursements totalling more than 100 million Euros since the implementation of DRG. Currently, approximately 2,000 up-coding generate an additional 20 million Euros per year (44).

Up-coding is the practice of classifying a patient in a DRG that results in a higher reimbursement or shifting a patient's DRG to another DRG that results in a higher payment from the third-party provider (25, 45). There are two primary methods for detecting potential DRG up-coding: (1) auditing by recoding the original medical charts, and (2) comparing historical claim data to detect an increase in the percentage of higher-cost DRGs (24).

Previous studies have indicated that DRG up-coding by private providers can be intentional (46). A code audit is the most reliable method of detecting DRG up-coding. Experienced health-information managers recode the original medical chart and then compare the new codes to the codes originally submitted by the hospital in code audit (46). Other research indicates that audits with fines can reduce up-coding while not necessarily inducing more honesty (47).

Another qualitative study on up-coding discovered that the Deliveries Group (2.8%) had the highest percentage of up-coding, followed by Female Reproductive System Groups (1.7%). Upcoding could result in a loss of IDR 154,626,000, or 9% of hospital revenue (48).

That study also discovered that the reasons for up-coding can be divided into three categories: (1) hospital-related; this occurred due to a lack of defined coding criteria. The hospital also did not know the flow of coordination between the teams constituted to tackle the problem of coding conflicts between the hospital and *BPJS Kesehatan*. (2) related to doctors; and (3) related to coders. Doctors frequently did not understand the coding standards. From the doctor's perspective, the disease's symptoms could also be incorporated into medical coding, but they couldn't. Furthermore, the coders occasionally have problems reading, and the doctor's handwriting and untranslated abbreviations are illegible (48).

To avoid human error in up-coding, doctors and coders should receive medical coding training to reduce doctor misspecifications or coder misunderstandings.

So far, the government's efforts in reducing moral hazard, including up-coding, have included the signing of an agreement or memorandum of understanding (MoU) between the *BPJS Kesehatan* and the Director of the Hospital, which includes the “Declaration of Absolute Responsibility Submission of Health Services Claims” and the “Statement of Claims by the Team Hospital Fraud Prevention,” which specifies that the hospital director is accountable for submitting claims files that are devoid of fraud or moral hazard. If there is fraud, the director is willing to face legal consequences.

This study discovered a low number of readmission incidents, with only 2.8%. Readmission cases were confirmed more in group P (Newborns and Neonates Group) and group W (Female reproductive system Groups) with two cases each.

Hospital readmissions can be defined as admissions to hospitals or other health care facilities arranged within a specific period of time following a hospital stay. Readmissions can also be defined as returning to the hospital within 30 days of being discharged (at first time), allowing the hospital to receive multiple reimbursements for the same treatment (12, 49, 50). The following factors contribute to readmissions: Inability to recognize the seriousness of the patient's illness, inability to appropriately address the patient's illness, patients being discharged from the hospital prematurely, and a lack of control over the hospital (12, 51, 52).

Furthermore, in research conducted by Auger et al. (53) on medical record review, 15% of readmissions were classified as unplanned and preventable. Researchers and policymakers concluded that a significant proportion of readmissions were caused by healthcare system failures—whether due to inadequate treatment during the initial hospitalization or a failure of care coordination after hospital discharge. Therefore, it is necessary to have policies to reduce inappropriate readmissions because hospitals receive additional payments when patients are readmitted (54).

Several interventions have succeeded in reducing readmission rates for discharged patients. These interventions include: patient needs assessment, medication reconciliation, patient education, timely outpatient appointments, and telephone follow-up. The impact of the intervention on the readmission rate is proportional to the number of components performed. This means that interventions with single component treatments are unlikely to reduce readmissions significantly (55).

Another finding in our study was physicians' age and specialization, coder's length of service, and LOS was the determinant factor of moral hazard in hospitals.

Older physicians are 1.037 times more likely than younger physicians to be associated with moral hazards (POR = 1.037). Other studies found that male physicians and older physicians were more likely to commit fraud, waste, and abuse on Medicare (56, 57). Based on the in-depth research conducted, it is stated, before beginning inpatient care, younger physicians learned the fundamentals of rules. As a result, their knowledge of coding rules keeps them more aware of moral hazards than older physicians. Older physicians may be resistant to new patient treatment rules, particularly coding rules that they believe are unfair to them. It is suggested that every CME (Continuing Medical Education) unit in the Faculty of Medicine should include Moral Hazard material in its activities in order to increase knowledge and skills, as well as develop doctors' attitudes so that they can always carry out their profession properly and correctly, and to help physicians understand the consequences of moral hazards and avoid them in the interest of their patient's health.

We also discovered that the specialization of physicians can influence the occurrence of moral hazards. Based on the study's findings, medical specializations are 2.373 times more likely to perform moral hazards rather than surgical, pediatrics, and OBGYN specializations (POR = 2.373). Previous research has found that the specialization of physicians influences the occurrence of fraud. According to Chen (57), physicians in certain specialties (such as family medicine, psychiatry, internal medicine, anesthesiology, surgery, and OBGYN) are more likely to commit Medicare fraud, waste, and abuse.

Pediatric specialization is used as the standard for calculating baselines in this study because the algorithm for compiling the INA-CBGs code in cases of pediatric is more complex, making manipulation difficult. According to this study, medical specialization is more likely to cause moral hazard than other specializations. Previous studies discovered Family medicine physicians and psychiatrists departed are more likely to commit fraud. This is because fraud is easier to commit when the risk of malpractice suits is very low, such as in the fields of family medicine and psychiatry. The study also explains that surgeons have the highest proportion of doctors who face malpractice claims based on their specialization (58).

*BPJS Kesehatan* is expected to be more stringent in inspecting cases of moral hazard to medical specialists and to continue to educate and raise awareness among physicians about the potential moral hazard in their medical practices.

We discovered that the length of service of the coder was a determinant of moral hazards. A beginner coder was 2.237 times more likely than an experienced coder to commit moral hazard (POR = 2.237). The length of service corresponds to the opportunity to receive training. Because the INA-CBG coding rules are not taught in detail in their studies, beginner coders have limitations in understanding the hospital coding regulations. Hospitals should provide regular training, particularly for new coders.

The LOS is the final factor discovered in this study that influences moral hazard in hospitals. Moral hazards are 1.249 times more likely to occur in long lengths of stay than short lengths of stay (POR = 1.249). Patients who require more treatment spend more days in hospitals. More hospital resources will be deployed as a result. Some hospitals are willing to take moral hazard, such as up-coding if they believe the INA-CBG tariff is insufficient for patient care. We recommend that the *BPJS Kesehatan* conduct more audits of hospitals with higher LOS.

The study's findings are highly encouraging, as it is well-known that senior physicians, medical specialists, coders with shorter lengths of service, and patients with longer lengths of stay. This discovery should encourage hospitals and insurance companies to be more cautious and pay more attention to audits of patient medical records containing these variable factors.

## 5. Strengths

In this study, each medical record was examined by an Independent Senior Coder (ISC). ISC comes from a higher-level hospital than the one being analyzed, where the coders are accustomed to coding more difficult and complex cases, and they have had national-level training more than five times.

## 6. Limitations

The operational definition of unnecessary admission in this study is a hospitalization of < 2 days, and discharges, which are not due to death. No doctor conducts a thorough examination of the unnecessary admission case. As a result, some cases of unnecessary admission may be considered necessary admission because they are not further evaluated by medical experts. The next study is expected to include a physician reviewing each case to determine whether hospitalization or admission is required.

## 7. Conclusion

This study revealed that the most common moral hazard is unnecessary admission, followed by up-coding and readmission. The factors significantly associated with moral hazard are physicians' age, physicians' specialization, coders' length of service, and LOS. The main factor that most has a role in moral hazard is the physician's specialization. It is suggested to the hospitals conduct training for physicians and coders about coding rules in casemix system in the hospital.

## Data availability statement

The raw data supporting the conclusions of this article will be made available by the authors, without undue reservation.

## Author contributions

SS, RM, SA, and RS contributed to conception and design of the study. SS organized the database and wrote the first draft of the manuscript. SS, RM, and SA performed the statistical analysis and wrote sections of the manuscript. All authors contributed to manuscript revision, read, and approved the submitted version.
